# *Ganoderma lucidum* stimulates autophagy-dependent longevity pathways in *Caenorhabditis elegans* and human cells

**DOI:** 10.18632/aging.203068

**Published:** 2021-05-20

**Authors:** Hsin-Hsin Peng, Cheng-Yeu Wu, Yuan-Chao Hsiao, Jan Martel, Po-Yuan Ke, Chen-Yaw Chiu, Jian-Ching Liau, I-Te Chang, Yu-Hsiu Su, Yun-Fei Ko, John D. Young, David M. Ojcius

**Affiliations:** 1 Center for Molecular and Clinical Immunology, Chang Gung University, Taoyuan, Taiwan; 2 Department of Traditional Chinese Medicine, Chang Gung Memorial Hospital at Linkou, Taoyuan, Taiwan; 3 Chang Gung Immunology Consortium, Chang Gung Memorial Hospital at Linkou, Taoyuan, Taiwan; 4 Research Center of Bacterial Pathogenesis, Chang Gung University, Taoyuan, Taiwan; 5 Department of Biochemistry and Molecular Biology, College of Medicine, Chang Gung University, Taoyuan, Taiwan; 6 Graduate Institute of Biomedical Sciences, College of Medicine, Chang Gung University, Taoyuan, Taiwan; 7 Liver Research Center, Chang Gung Memorial Hospital at Linkou, Taoyuan, Taiwan; 8 Division of Allergy, Immunology and Rheumatology, Chang Gung Memorial Hospital at Linkou, Taoyuan, Taiwan; 9 Biochemical Engineering Research Center, Ming Chi University of Technology, New Taipei City, Taiwan; 10 Chang Gung Biotechnology Corporation, Taipei, Taiwan; 11 Department of Biomedical Sciences, University of the Pacific, Arthur Dugoni School of Dentistry, San Francisco, CA 94103, USA

**Keywords:** caloric restriction mimetics, dietary supplements, lingzhi, medicinal mushrooms, mTOR

## Abstract

The medicinal fungus *Ganoderma lucidum* is used as a dietary supplement and health tonic, but whether it affects longevity remains unclear. We show here that a water extract of *G. lucidum* mycelium extends lifespan of the nematode *Caenorhabditis elegans*. The *G. lucidum* extract reduces the level of fibrillarin (FIB-1), a nucleolar protein that correlates inversely with longevity in various organisms. Furthermore, *G. lucidum* treatment increases expression of the autophagosomal protein marker LGG-1, and lifespan extension is abrogated in mutant *C. elegans* strains that lack *atg-18*, *daf-16*, or *sir-2.1*, indicating that autophagy and stress resistance pathways are required to extend lifespan. In cultured human cells, *G. lucidum* increases concentrations of the LGG-1 ortholog LC3 and reduces levels of phosphorylated mTOR, a known inhibitor of autophagy. Notably, low molecular weight compounds (<10 kDa) isolated from the *G. lucidum* water extract prolong lifespan of *C. elegans* and the same compounds induce autophagy in human cells. These results suggest that *G. lucidum* can increase longevity by inducing autophagy and stress resistance.

## INTRODUCTION

Aging is a malleable process that can be modulated by genes, diet and lifestyle [1]. For instance, rare mutations and single nucleotide polymorphisms (SNPs) in pathways involving forkhead box O3A (FOXO3A) and insulin-like growth factor-1 (IGF-1) receptor are associated with extended lifespan in centenarians [2, 3]. Caloric restriction (CR) also extends lifespan in various species including yeasts, nematodes, fruit flies, rodents and monkeys [4–6]. Given that animals fed CR diets usually consume their daily allocated food rapidly and in a single serving, the effects of CR may in reality be due to long periods of food abstinence (>16 hrs/day; i.e., intermittent fasting) [5, 7]. Similarly, exercise improves health markers and is widely believed to improve the healthspan and lifespan in humans [8]. Many plant and fungal compounds found in the diet, such as polyphenols, terpenoids and alkaloids also extend lifespan in model organisms and produce health benefits in humans [9–12].

Anti-aging interventions improve health and longevity by activating stress resistance pathways in the host via hormesis, which posits that low amounts of intermittent stress can improve cellular functions and produce health benefits, while higher levels of stress are detrimental [11, 13–15]. The organism adapts in response to mild stress by reducing the amount of energy and resources allocated to growth and reproduction, and instead uses the limited resources to improve cell maintenance and survival [16]. Accordingly, CR, intermittent fasting, exercise and phytochemicals activate cellular pathways that induce autophagy, DNA repair, mitochondrial biogenesis and expression of antioxidant and detoxifying enzymes, which together improve cellular and organ functions [4, 7, 17]. Notably, autophagy plays a critical role in the anti-aging interventions identified to date by degrading damaged proteins and organelles in a process that is analogous to cellular recycling [18].

Given that the anti-aging lifestyle interventions may be difficult to implement on a daily basis, considerable interest has been devoted to identifying molecules that induce autophagy and promote longevity (i.e., CR mimetics). Many candidate compounds in this category have been isolated or derived from natural sources such as aspirin, metformin, rapamycin, glucosamine, polyphenols, and spermidine [10, 19]. Since CR mimetics such as aspirin and rapamycin can produce unwanted side effects, identification of new and safe CR mimetics is needed. In this context, *Caenorhabditis elegans* represents a useful model for identifying CR mimetics from natural health products due to its short lifespan, ease of manipulation, and tractable genetics [20], even though many precautions need to be considered [21].

*Ganoderma lucidum* (GL), also known as lingzhi or reishi, is a fungus with a long history of use in Asia as a tonic to improve health and vitality [22]. Recent research shows that GL produces various health benefits in animal models, including anti-inflammatory, anti-diabetic and anti-cancer effects [23, 24]. While screening for bioactive compounds derived from medicinal fungi, we observed that high molecular weight polysaccharides isolated from GL mycelia reduced obesity, inflammation, insulin resistance and fatty liver disease in mice fed with a high-fat diet [25]. Similarly, another study showed that polysaccharides isolated from GL extended the lifespan of *C. elegans* through a process dependent on *daf-16* [26], the ortholog of the human FOXO transcription factors involved in stress resistance. Finally, another report demonstrated that a water extract of GL prolonged the lifespan of *C. elegans* by producing antioxidant effects and modulating germline signaling [27]. Nonetheless, the mechanisms whereby GL compounds may affect aging in animal models and human cells remain poorly understood.

In the present study, we showed that a water extract of GL and a sub-fraction containing polysaccharides, oligosaccharides and other low-molecular-weight compounds promote longevity in nematodes by inducing autophagy. Notably, GL and the same sub-fraction also induce autophagy in human cells, indicating that the effects produced by the fungus may be conserved across different species.

## RESULTS

### *G. lucidum* reduces FIB-1 protein levels in nematodes

**Figure 1:**
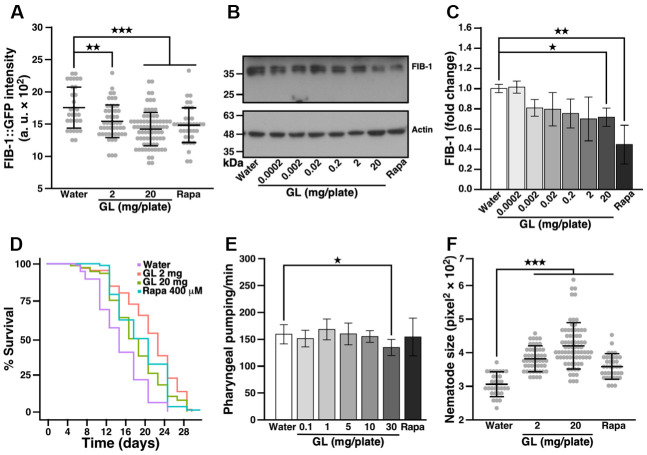
***G. lucidum* treatment reduces FIB-1 levels and extends lifespan in *C. elegans*.** (**A**) Effects of a water extract of *G. lucidum* (GL) on fibrillarin-1 (FIB-1) levels as monitored by fluorescence microscopy. Synchronized L4 larvae of transgenic *C. elegans* strain SJL1 expressing FIB-1::GFP (green fluorescent protein) under the FIB-1 gene’s native promoter were cultured for 3 days on agar plates spread with control water, the water extract of GL, or rapamycin (400 μM, Rapa). Data are expressed as arbitrary units (a. u.). (**B**) FIB-1 levels assessed by Western blots in wild-type N2 *C. elegans* treated with GL. (**C**) Quantification of FIB-1 protein levels shown in (**B**). FIB-1 expression was measured by densitometry and normalized against actin. (**D**) Lifespan assay of GL-treated nematodes. *C. elegans* was cultured on agar plates spread with water, GL or rapamycin as above. Survival was assessed for 30 days using an optical microscope based on motility. Representative lifespan curves are shown. See also Supplementary Table 1. (**E**) Pharyngeal pumping of nematodes following culture with water, GL or rapamycin for 3 days. Pharyngeal contractions were recorded for 1 min under optical microscopy. (**F**) Size of 3-day old worms. Size was monitored by delineating the worms’ region of interest (ROI) under optical microscopy. Data represent means ± standard deviation. *p<0.05; **p<0.01; ***p<0.001.

Earlier work showed that rRNA synthesis and nucleolar size are inversely correlated with lifespan in *C. elegans*, fruit flies, mice, and humans [28]. Accordingly, the abundance of the fibrillarin protein FIB-1—a nucleolar rRNA 2’-O-methyltransferase involved in processing of pre-rRNA—negatively correlates with lifespan and can thus be used as a marker to study longevity [28]. We used a transgenic nematode strain (SJL1) described earlier [20, 29] which expresses a FIB-1 construct coupled with green fluorescent protein (GFP; Supplementary Figure 1A, 1B) to identify natural products that affect aging. Under fluorescence microscopy, we observed that GL reduced FIB-1::GFP intensity in a dose-dependent manner, compared with water control (Figure 1A). GL also reduced FIB-1 protein levels in wild-type N2 nematodes as assessed by Western blotting (Figure 1B, 1C). As a positive control, rapamycin also reduced FIB-1 protein levels in these assays (Figure 1A–1C), consistent with the anti-aging effects produced by this compound in various organisms [18].

### *G. lucidum* extends lifespan in *C. elegans*

To determine whether *G. lucidum* can influence longevity, we measured the effects of GL on the lifespan of *C. elegans*. In these experiments, nematodes were fed with UV-killed *Escherichia coli* to prevent the possibility that GL may affect bacterial growth and energy intake. Consistent with the results shown above for FIB-1, GL used at 2 mg/plate extended median lifespan of *C. elegans* from 13.0 ± 2.8 to 18.5 ± 6.3 days compared to control water, representing a 45% extension (Figure 1D and Supplementary Table 1). In this case, the low GL concentration (2 mg/plate) was more effective than the high concentration (20 mg/plate) (Figure 1D and Supplementary Table 1), consistent with a hormetic dose response [11, 15]. Notably, the lifespan extension produced by GL (2 mg/plate) was more pronounced than the extension induced by rapamycin, which extended median lifespan to 16.0 ± 2.8 days (Figure 1D and Supplementary Table 1). GL also prolonged maximum lifespan from 18.8 ± 1.6 to 28.9 ± 2.3 days (Figure 1D and Supplementary Table 1, 20 mg/plate). Of note, treatment with GL did not affect pharyngeal pumping (Figure 1E, up to a concentration of 10 mg/plate), indicating that the extract did not extend lifespan by reducing food intake. In control experiments, both GL and rapamycin increased the number of nematodes that moved out of the bacterial lawn containing the tested substance (Supplementary Figure 1C). In addition, nematodes fed GL were larger than controls (Figure 1F), while their triglyceride content was reduced (Supplementary Figure 1D), producing changes similar to rapamycin (Figure 1F and Supplementary Figure 1D).

### *G. lucidum* extends lifespan by inducing autophagy

**Figure 2:**
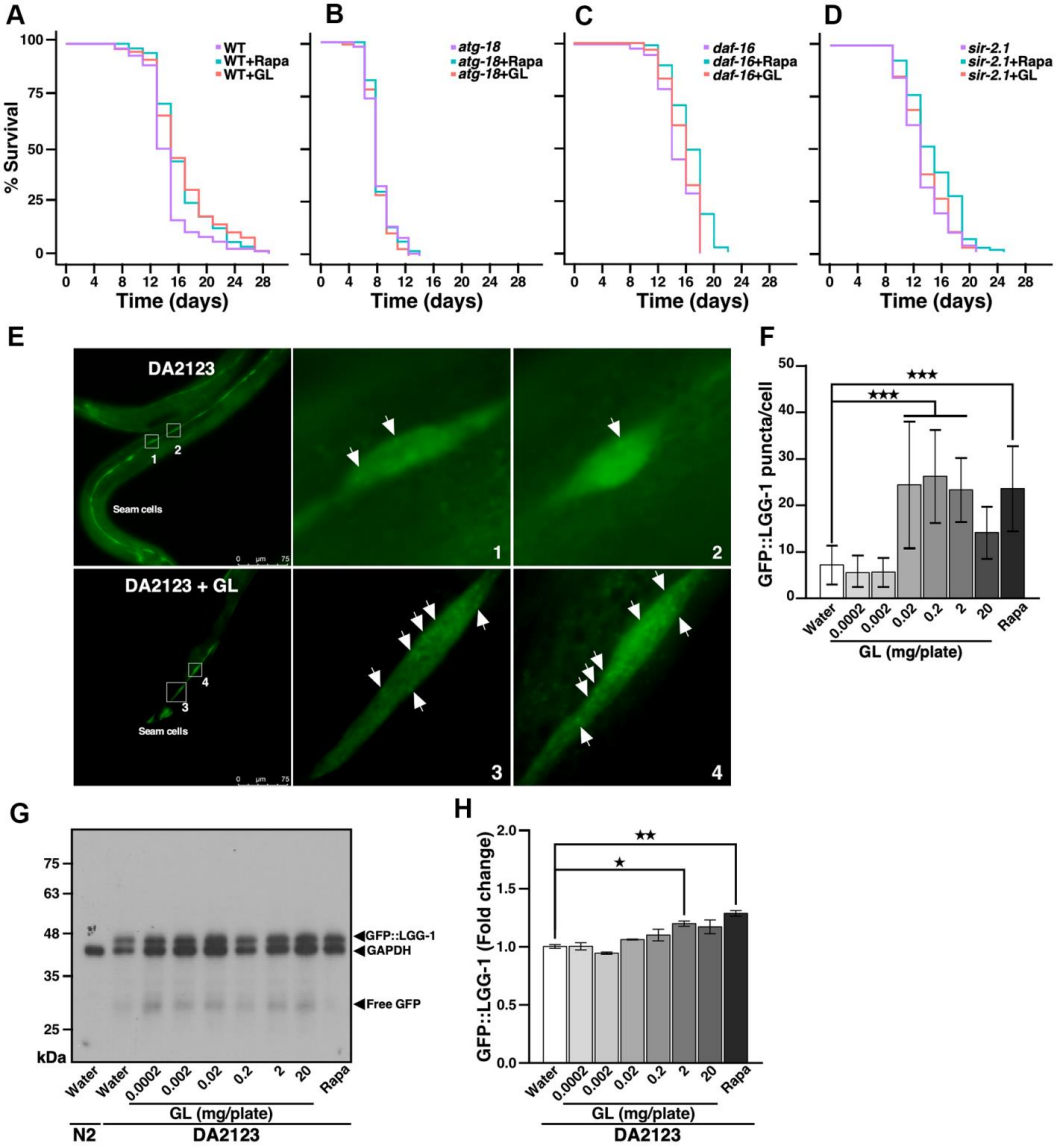
***G. lucidum* extends nematode lifespan by inducing autophagy.** (**A**–**D**) Effects of *G. lucidum* (GL) in wild-type and mutant *C. elegans*. Synchronized L4 larvae of (**A**) wild-type (WT) N2 *C. elegans* or mutant strains lacking (**B**) *atg-18*, (**C**) *daf-16*, or (**D**) *sir-2.1* were maintained on agar plates spread with GL (2 mg/plate) or rapamycin (Rapa, 400 μM), and survival was monitored based on motility (see also Supplementary Table 1). (**E**) GL induces autophagy in *C. elegans*. Transgenic DA2123 *C. elegans* expressing GFP::LGG-1 were treated as above for 3 days, prior to observation under fluorescence microscopy. Approximately 50 cells were examined per treatment. (**F**) Quantification of fluorescent GFP::LGG-1 puncta following GL treatment based on the experiments shown in (**E**). (**G**) GL treatment increases GFP::LGG-1 levels in DA2123 worms as revealed by Western blotting. Membranes were incubated with both anti-GFP and anti-glyceraldehyde 3-phosphate dehydrogenase (GAPDH) antibodies, prior to signal detection. (**H**) Quantification of Western blot signals shown in (**G**) after normalization against GAPDH. *p<0.05; **p<0.01; ***p<0.001.

Given that CR mimetics can usually activate autophagy [19], we examined the possibility that GL may extend lifespan by inducing this cellular process. We tested the effects of GL in mutant nematodes that lack *atg-18*, which encodes a protein required for autophagy [18]. While GL extended lifespan in wild-type N2 nematodes (Figure 2A and Supplementary Table 1), no lifespan extension was observed in nematodes lacking *atg-18* (Figure 2B and Supplementary Table 1), indicating that autophagy is required for lifespan extension. Furthermore, GL did not extend lifespan in mutant worms lacking either *daf-16* or *sir-2.1* (Figure 2C, 2D, and Supplementary Table 1). Moreover, GL increased expression of the protein LGG-1 (Figure 2E–2H), the ortholog of mammalian light chain 3 (LC3), which is involved in autophagy [18]. That is, transgenic DA2123 nematodes expressing GFP::LGG-1 showed higher numbers of LGG-1^+^ puncta following GL treatment compared to water-treated controls in which fluorescence was diffuse (Figure 2E, 2F). In these experiments, a hormetic dose response involving peak stimulation of autophagy at low concentrations (0.2 mg/plate) and lower levels of activation at high concentrations (20 mg/plate) was also observed (Figure 2F). Western blotting assays also revealed that GL treatment increased GFP::LGG-1 levels in transgenic DA2123 nematodes in a manner like rapamycin (Figure 2G, 2H). These results indicate that *G. lucidum* prolongs lifespan by activating autophagy and stress resistance pathways in nematodes.

### *G. lucidum* induces autophagy in human cells

**Figure 3:**
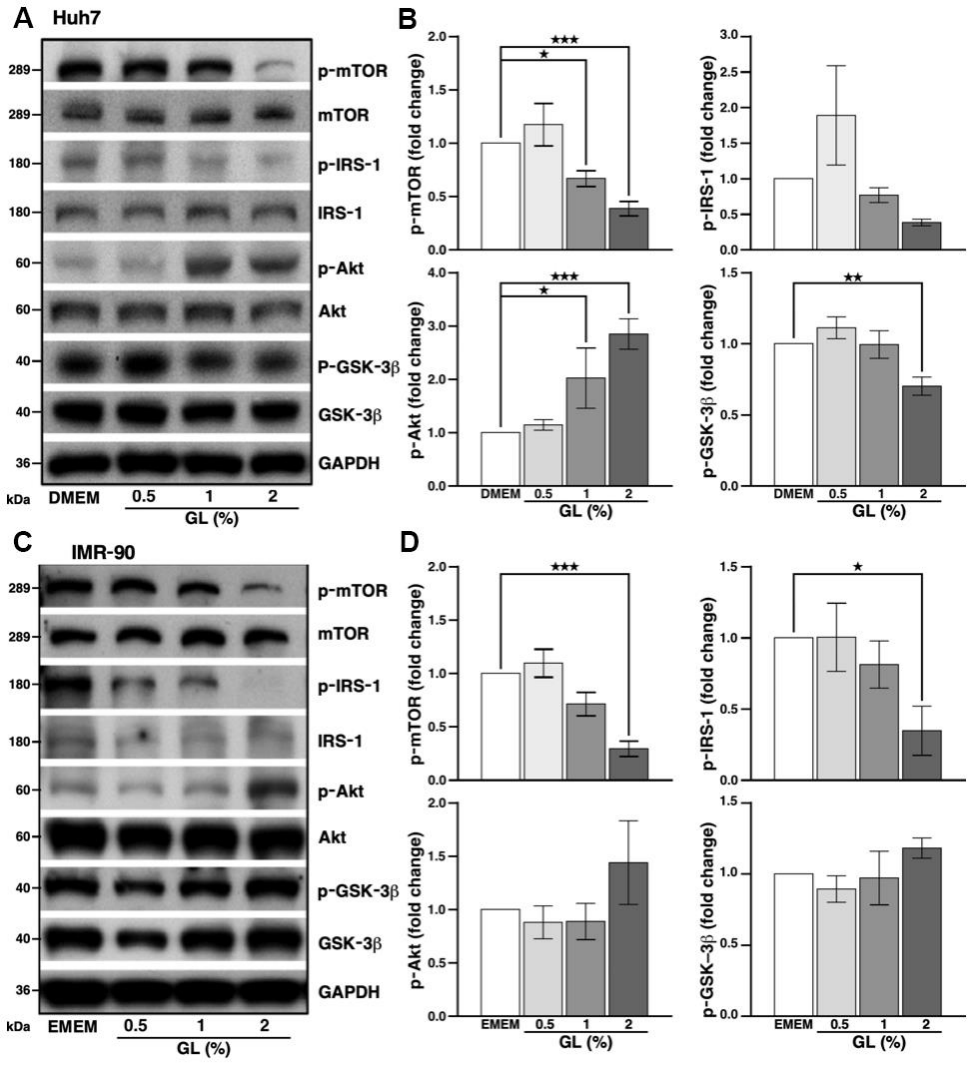
***G. lucidum* inhibits the mTOR pathway in human cells.** (**A**) Effects of *G. lucidum* (GL) on the mTOR pathway in human Huh7 hepatoma cells. Cells were treated with GL for 4 hrs, prior to Western blot analysis. Expression was normalized against GAPDH. (**B**) Protein intensity evaluated by densitometry. (**C**, **D**) Effects of GL on the mTOR pathway in human IMR-90 lung fibroblasts. Cells were processed as above for (**C**) Western blotting and (**D**) densitometry analysis. Statistical analysis was done with one-way analysis of variance (ANOVA). *p<0.05; **p<0.01; ***p<0.001.

We tested whether GL induced autophagy in the human hepatoma Huh7 cell line, which is routinely used to assess this cellular process. Cell viability assays showed that GL did not affect the viability of Huh7 liver cells at doses of 0.5 to 2% (Supplementary Figure 2A). For IMR-90 lung fibroblasts, a non-cancerous cell line often used in aging-related studies, GL did not affect viability at doses of 0.5 and 1%, but cytotoxic effects were observed at 2% (Supplementary Figure 2C). Western blot analysis performed on Huh7 cells showed that GL decreased the level of phosphorylated mTOR (p-mTOR; Figure 3A, 3B), which acts as a repressor of autophagy [18]. Similarly, GL at a dose of 2% slightly reduced the phosphorylation of insulin receptor substrate-1 (p-IRS-1) (Figure 3A, 3B), an upstream activator of mTOR [18]. Furthermore, GL reduced phosphorylated GSK-3β (p-GSK-3β), but increased phosphorylation of Akt on serine 473 (p-Akt; Figure 3A, 3B), possibly due to a negative feedback mechanism, as observed earlier in various cancer cell lines treated with mTOR inhibitors such as everolimus [30] and rapamycin [31]. Similar results were obtained for p-mTOR and p-IRS-1 in IMR-90 fibroblasts, although no statistically significant changes were observed for p-Akt and p-GSK-3β in these cells (Figure 3C, 3D).

**Figure 4:**
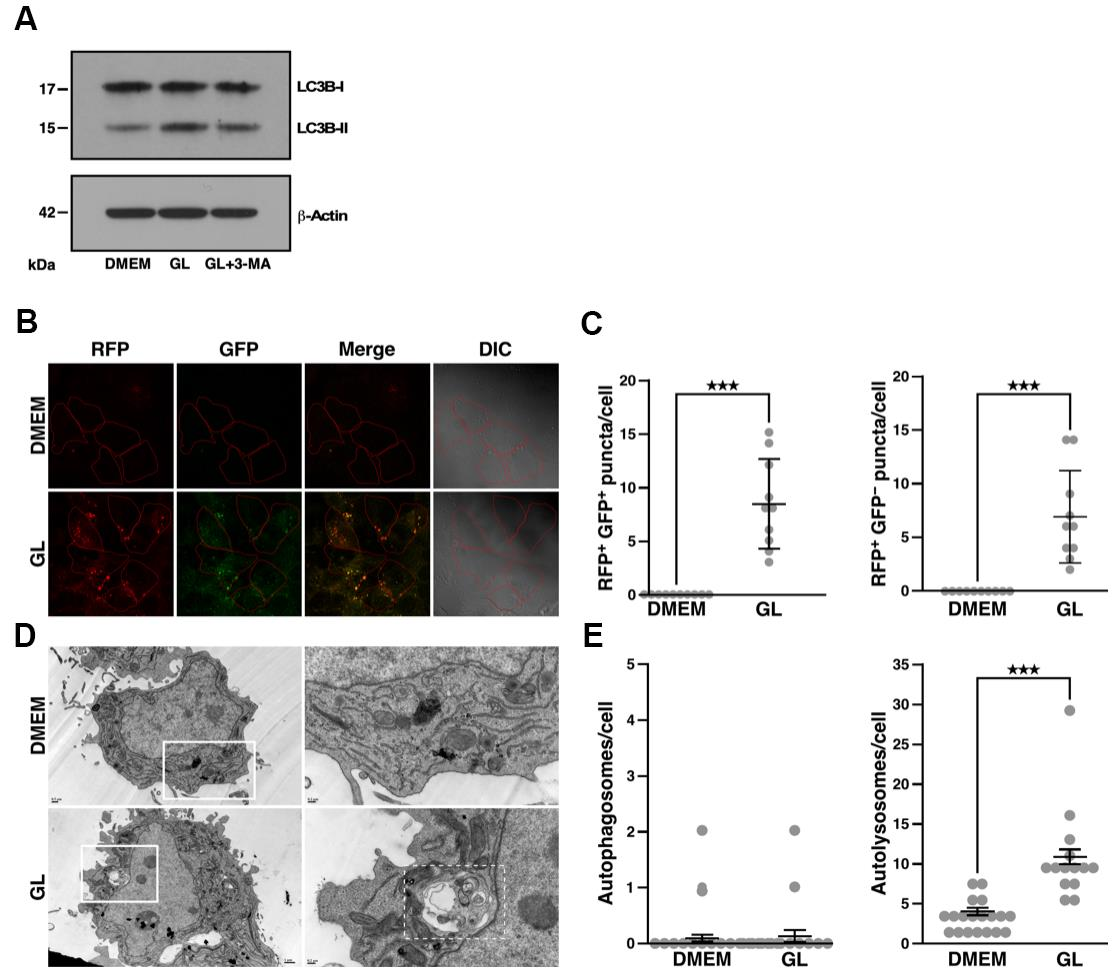
***G. lucidum* induces autophagy in human cells.** (**A**) Effects of *G. lucidum* (GL) on LC3B-I and LC3B-II in human cells. Huh7 cells maintained in Dulbecco’s modified Eagle’s medium (DMEM) were treated with GL (1%) or DMEM for 12 hrs, prior to treatment with DMEM containing 3-methyladenine (3-MA, 2 mM) for 12 hrs. Protein levels were monitored by Western blot and normalized against actin. (**B**) GL induces the formation of autophagosomes and autolysosomes in human cells. Huh7 cells expressing monomeric red fluorescent protein (RFP)-LC3 and green fluorescent protein (GFP)-LC3 were treated with GL (1%) for 24 hrs, prior to fluorescence microscopy analysis. In differential interference contrast (DIC) images, cells are delineated in red for clarity. (**C**) Quantification of fluorescent puncta based on fluorescence microscopy. (**D**) Representative transmission electron microscopy (TEM) images of GL-treated cells. Huh7 cells were treated with GL (1%) for 24 hrs prior to fixation and thin-sectioning as described in *Materials and Methods*. Images on the right correspond to the insets delineated by white rectangles in the images on the left. An autolysosome is delineated by a white dashed line for the GL panel. (**E**) Quantification of autophagosomes and autolysosomes based on TEM. ***p<0.001.

Treatment of Huh7 cells with GL increased conversion of LC3B-I to LC3B-II (Figure 4A), which is used as a marker to monitor autophagy activation. As expected, treatment with the autophagy inhibitor, 3-methyladenine, reduced GL-induced LC3B-II protein accumulation (Figure 4A). On the other hand, GL did not affect the phosphorylation of other autophagy-related proteins, such as Beclin-1 and p38/p44/42 mitogen-activated protein kinases (MAPKs; Supplementary Figure 3A, 3B). Using Huh7 cells that express monomeric red fluorescent protein (RFP)-LC3 and GFP-LC3 [32], we observed that GL induced the formation of autophagosomes (consisting of RFP^+^ GFP^+^ puncta) and autolysosomes (RFP^+^ GFP^–^ puncta, as GFP-LC3 fluorescence is quenched in autolysosomes) (Figure 4B, 4C; see also Supplementary Figure 3C, 3D). Similar results were obtained in IMR-90 lung fibroblasts stained with dyes that label autolysosomes and autophagosomes (Supplementary Figure 4A, 4B). Notably, GL also increased the number of autolysosomes as revealed by transmission electron microscopy (TEM), while the number of autophagosomes was not significantly affected in this case (Figure 4D, 4E). These observations indicate that GL can induce autophagy in cultured human cells.

### Effects of *G. lucidum* sub-fractions in *C. elegans* and human cells

**Figure 5:**
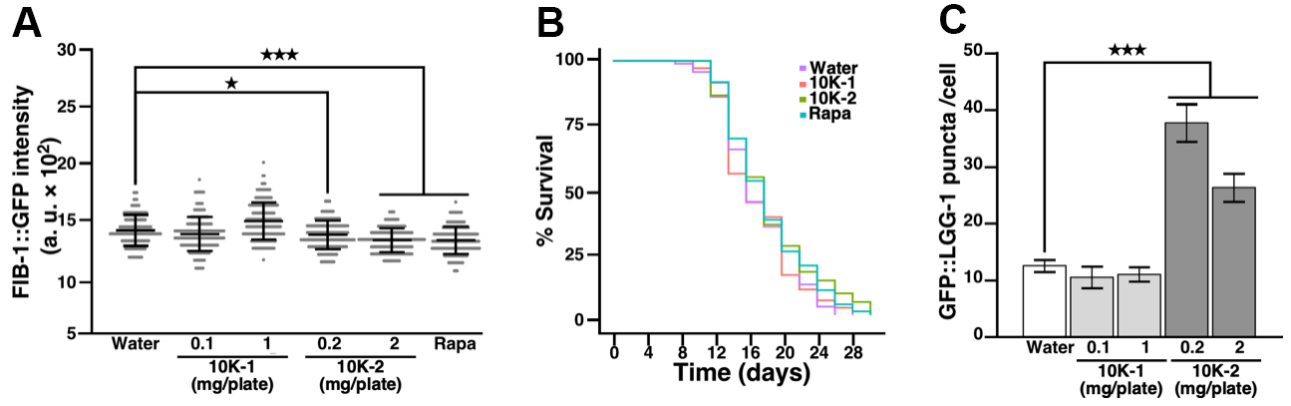
***G. lucidum*-derived subfraction 10K-2 attenuates FIB-1 expression, extends lifespan, and induces autophagy in *C. elegans*.** (**A**) Effects of *G. lucidum* (GL) sub-fractions on FIB-1::GFP intensity. Synchronized L4 larvae of *C. elegans* SJL1 were treated with water, GL sub-fractions, or rapamycin (Rapa; 400 μM) for 3 days. FIB-1::GFP was monitored by fluorescence microscopy. (**B**) Lifespan assay of *C. elegans* treated with GL sub-fractions. Synchronized *C. elegans* SJL1 larvae were treated with water, GL subfractions 10K-1 (1 mg/plate) or 10K-2 (2 mg/plate), or rapamycin (400 μM). Representative survival curves are shown (see also Supplementary Table 1). (**C**) GL sub-fraction 10K-2 induces autophagy in *C. elegans*. DA2123 nematodes were treated with the sub-fractions for 3 days, and GFP::LGG-1 levels were quantified by fluorescence microscopy. Numbers of GFP puncta were counted in 50 seam cells. Data represent means ± standard deviation. Statistical analysis was done using Student’s t test. *p<0.05; ***p<0.001.

We used ultrafiltration to prepare sub-fractions containing polysaccharides, oligosaccharides and small compounds from GL (Supplementary Tables 2–5), and tested the ability of these sub-fractions to modulate FIB-1::GFP protein levels in transgenic SJL1 *C. elegans*. Sub-fraction 10K-1 containing high molecular weight compounds and polysaccharides (>10 kDa) did not affect FIB-1::GFP protein levels in the treated nematodes, whereas sub-fraction 10K-2 containing polysaccharides, oligosaccharides and other compounds of low molecular weight (<10 kDa) significantly reduced FIB-1::GFP protein levels, producing effects similar to that of rapamycin (Figure 5A). In lifespan assays, sub-fraction 10K-2 (2 mg/plate) extended median lifespan in nematodes, whereas 10K-1 did not produce statistically significant results (Figure 5B and Supplementary Table 1).

Consistent with these results, sub-fraction 10K-2 increased levels of GFP::LGG-1 in transgenic DA2123 nematodes, while sub-fraction 10K-1 had no effect (Figure 5C).

**Figure 6:**
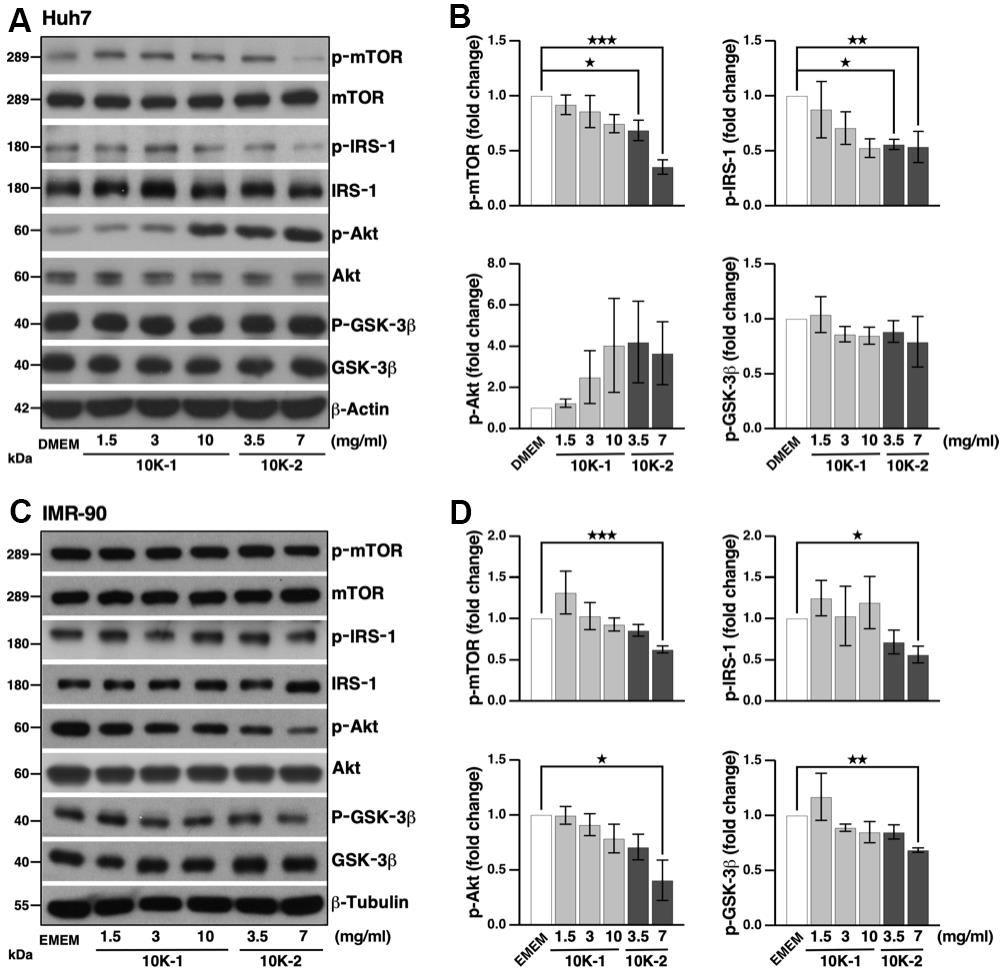
***G. lucidum*-derived sub-fraction 10K-2 represses the mTOR pathway in human cells.** (**A**) Inhibition of the mTOR pathway by *G. lucidum* (GL) sub-fraction 10K-2. Huh7 liver cells were treated with control medium (i.e., Dulbecco’s modified Eagle’s medium, DMEM), 10K-1 or 10K-2 for 4 hrs, prior to Western blot analysis. (**B**) Protein intensity was evaluated by densitometry and normalized against actin. (**C**) Sub-fraction 10K-2 inhibits the mTOR pathway in IMR-90 cells. Cells cultured in Eagle’s miminum essential medium (EMEM) were processed as above for Western blot analysis. (**D**) Densitometry analysis of the results shown in (**C**). *p<0.05; **p<0.01; ***p<0.001.

**Figure 7:**
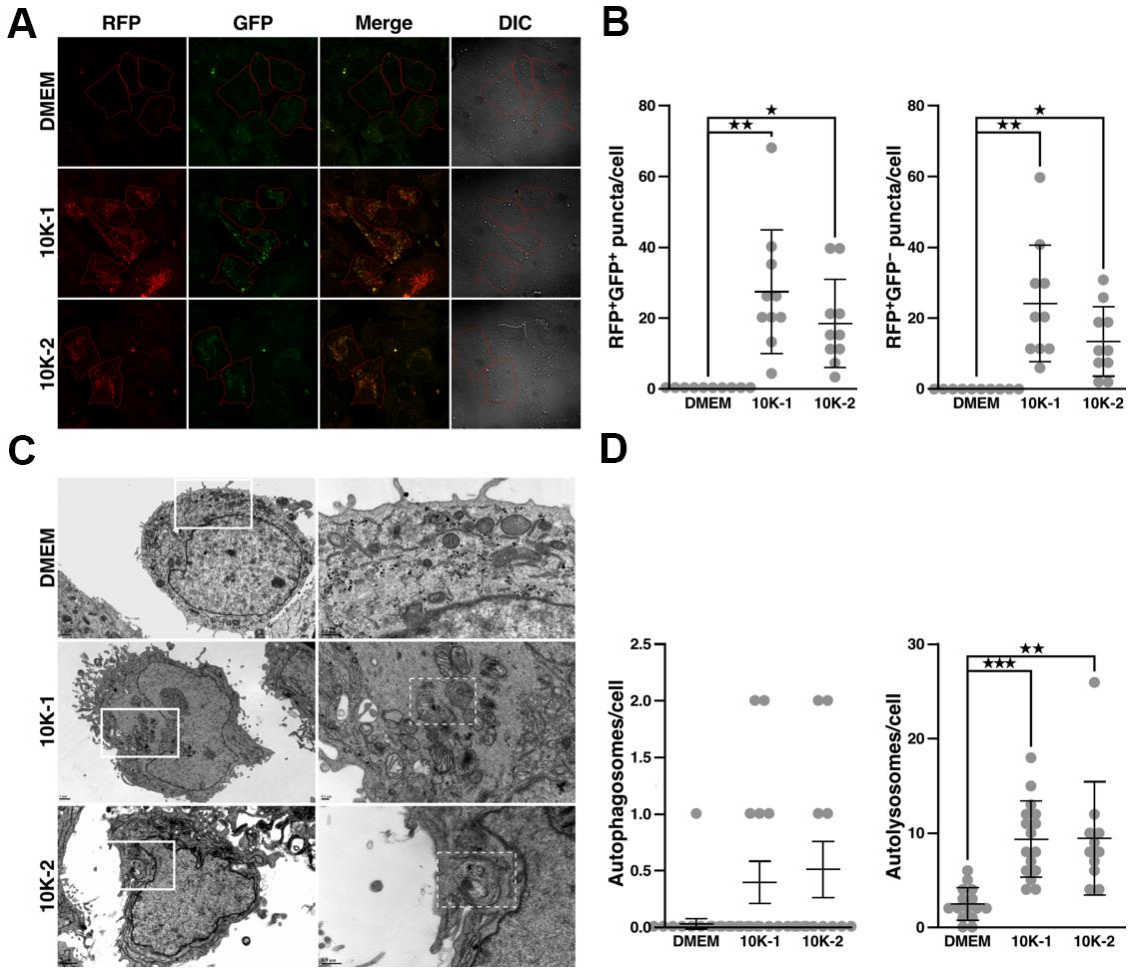
***G. lucidum*-derived sub-fraction 10K-2 induces autophagy in human cells.** (**A**) *G. lucidum* (GL) sub-fractions 10K-1 and 10K-2 induce the formation of autophagosomes and autolysosomes in human cells. Huh7 cells expressing mRFP-GFP-LC3 were treated with DMSO or GL sub-fractions 10K-1 and 10K-2 (1 mg/ml) for 24 hrs, prior to fluorescence microscopy observations. (**B**) Quantification of fluorescent puncta based on fluorescence microscopy analysis. (**C**) TEM observations of Huh7 cells treated with GL sub-fractions. Cells were treated as above prior to fixation and preparation for TEM analysis. Images on the right correspond to the white rectangles in the images on the left. Autolysosomes are delineated by a white dashed line in the 10K-1 and 10K-2 panels. (**D**) Quantification of autophagosomes and autolysosomes based on TEM analysis. *p<0.05; **p<0.01; ***p<0.001.

In human cells, sub-fractions 10K-1 and 10K-2 did not have a negative effect on cell viability (Supplementary Figure 2B, 2D). Sub-fraction 10K-2 reduced levels of p-mTOR and p-IRS-1 in Huh7 cells, while p-Akt and p-GSK-3β were unaffected (Figure 6A, 6B). In IMR-90 fibroblasts, sub-fraction 10K-2 reduced p-mTOR, p-IRS-1, p-Akt, and p-GSK-3β levels (Figure 6C, 6D). Notably, sub-fraction 10K-2 induced autophagy in human cells, as revealed by the increased number of autophagosomes (RFP^+^ GFP^+^ puncta) and autolysosomes (RFP^+^ GFP^–^ puncta; Figure 7A, 7B; see also Supplementary Figure 3E, 3F). These results were confirmed in IMR-90 lung fibroblasts stained with dyes that label autolysosomes and autophagosomes (Supplementary Figure 4A, 4B).

Similarly, 10K-2 increased autolysosomes in Huh7 cells based on TEM analysis (Figure 7C, 7D). Sub-fraction 10K-1 also induced autophagy in some of these experiments (Figure 7A–7D, and Supplementary Figure 3E, 3F), but mTOR signaling was not affected in this case (Figure 6A–6D).

## DISCUSSION

While *G. lucidum* is known to possess various bioactivities in cultured cells and animal models, few studies had evaluated how this medicinal fungus may affect aging and lifespan. We show here that GL and sub-fraction 10K-2 containing polysaccharides, oligosaccharides and possibly other compounds of low molecular weight extend lifespan in nematodes by inducing autophagy and stress resistance. Autophagy can improve cellular functions by reducing the amount of damaged proteins and organelles, while stress resistance pathways involving *daf-16* and *sir-2.1* can produce benefits by inducing expression of heat-shock proteins as well as antioxidant and detoxifying enzymes [20]. Our results are consistent with previous studies showing that *G. lucidum* water extract can extend lifespan in *C. elegans* [26, 27]. Notably, Cuong et al. showed that a water extract of *G. lucidum* extended lifespan by producing antioxidant effects and by modulating the mTOR/S6K and germline signaling pathways [27]. Many of the beneficial effects produced by *G. lucidum* in previous studies may be attributed to induction of autophagy and stress resistance mechanisms in a manner similar to phytochemicals from fruits and vegetables [17].

We observed that low doses of GL and sub-fraction 10K-2 (and to a lesser extent 10K-1) can induce autophagy in *C. elegans*, while high doses are less efficient at inducing this process (see for instance Figures 2F, 5C). These observations are consistent with the hormesis dose response, which is a general response produced by phytochemicals and fungal chemicals [11, 13, 14]. This dose response may have been overlooked in previous studies, possibly due to the large range of concentrations needed to observe the opposite effects [15]. In addition, previous studies showed that *G. lucidum* can either induce or inhibit autophagy in cancer cells [33, 34]. Cancer cells may therefore also react in a hormesis-dependent manner to cellular stress produced by *G. lucidum* compounds such as polysaccharides, as described in previous studies [35].

The *G. lucidum* extract and sub-fractions studied here extend nematode lifespan and inhibit mTOR signaling in human cells in a manner similar to rapamycin, a compound that is being considered to delay aging in healthy humans [36]. However, the possible side effects induced by rapamycin including immunosuppression and insulin resistance may limit the use of this compound as a CR mimetic in healthy humans. Fungal polysaccharides represent good candidates for use as CR mimetics as they are not digested by human digestive enzymes; they are poorly absorbed in the human digestive tract; they may inhibit digestive enzymes and lipid absorption; they produce only minor side effects, if any; and they provide minimal calories from the short-chain fatty acids produced by the gut microbiota [17, 37]. Consistent with our results, mushroom extracts that contain polysaccharides are found in the fasting-mimicking diet designed by Longo et al. which has been shown to produce various health benefits in animal models, including anti-diabetic, anti-cancer and neuroprotective effects [38–41]. Testing in mice and humans will be needed to confirm the possibility that fungal polysaccharides can be used as CR mimetics to improve health and longevity in higher organisms. However, we cannot exclude the possibility that other uncharacterized compounds may also contribute to the anti-aging effects produced by the *G. lucidum* extract and sub-fractions studied here.

Various lifestyle interventions such as CR, intermittent fasting and exercise are being considered to delay aging and treat chronic diseases. However, these interventions can be difficult to implement in the general population, an observation which prompted interest in the development of CR mimetic compounds. Our data suggest that *G. lucidum* polysaccharides and oligosaccharides are potential candidates to serve as CR mimetics. Further testing will show whether *G. lucidum* can extend lifespan and induce autophagy in animal models.

## MATERIALS AND METHODS

### Preparation of *G. lucidum* water extract and sub-fractions

*G. lucidum* mycelium was isolated and characterized at Chang Gung Biotechnology Corporation from a specimen isolated in northern Taiwan. Species identification was confirmed by sequencing 5.8S rDNA and internal transcribed spacers as before [42]. The water extract of GL was prepared from cultured mycelium as described [42]. Briefly, 40 g of dried *G*. *lucidum* mycelium was mixed with 800 ml of double distilled water and the solution was heated at 121° C for 45 min. The solution was centrifuged at 5,900×*g* for 20 min and the supernatant was processed with a vacuum concentrator. The powder obtained was dissolved in water to obtain a final concentration of 20% (w/v). The GL solution was autoclaved for 20 min and stored at 4° C.

Sub-fractions 10K-1 and 10K-2 were prepared from a 5% (w/v) solution of GL water extract using a KrosFlo tangential flow filtration system with a 10-kDa molecular weight cutoff membrane (Spectrum Laboratories). Transmembrane pressure was maintained at 8–10 pounds per square inch (PSI). Sub-fraction 10K-1 (>10 kDa) was gathered by repeatedly washing the membrane retentate following filtration, while 10K-2 (<10 kDa) was collected as the filtrate. Specimens were processed with a vacuum freeze drier and stored at 4° C. Monosaccharide composition was determined using high-performance ion chromatography with pulsed amperometric detection (Dionex ICS-5000, Thermo Scientific). Protein content was analyzed using the Bradford assay (Bio-Rad). Molecular weight analysis was done with gel permeation chromatography (Viscotek SEC-MALS 20, Malvern Panalytical). Analyses were performed based on the manufacturers’ guidelines.

### Nematode strains and culture

The *C. elegans* wild-type strain N2 and the transgenic strain SJL1 expressing a FIB-1::GFP plasmid construct [29] were kindly provided by Dr. Szecheng J. Lo (Chang Gung University). Transgenic *C. elegans* strains DA2123 (expressing a GFP::LGG-1 plasmid construct), VC893 (lacking *atg-18*), CF1038 (lacking *daf-16*), and VC199 (lacking *sir-2.1*) were purchased from the *C. elegans* Core Facility Taiwan. Nematodes were maintained at 20° C on nematode growth medium (NGM; 2% agar, 0.5% bactopeptone, 50 mM NaCl, 25 mM potassium phosphate buffer, 1 mM CaCl_2_, 1 mM MgSO_4_, 5 μg/ml cholesterol, pH 6) with UV-killed *E. coli* strain OP50 as food source.

Synchronization of nematode growth was done by bleaching and the egg laying method. Briefly, nematodes were grown onto NGM plates and gravid adults were collected in 15-ml tubes by washing plates with phosphate buffered saline (PBS) containing Tween-20 (PBST buffer). Adult nematodes were washed by centrifugation at 400×*g* for 2 min, followed by resuspension in 600 μl of bleaching solution (450 μl of PBST buffer, 100 μl of 6% bleach, 50 μl of 10 M KOH) and agitation for 10 min. The bleaching reaction was stopped by adding 10 ml of PBST buffer and the solution was centrifuged at 400×*g* for 1 min to pellet eggs, which were washed three times in PBST as above. Washed eggs were resuspended in 1 ml of PBST buffer and inoculated onto NGM plates, prior to incubation at 20° C. After overnight incubation, synchronized nematode larvae were transferred to NGM plates spread with *E. coli* OP50, rapamycin (400 μM), GL (0.0002–30 mg/plate), and/or subfractions 10K-1 and 10K-2 (0.1–2 mg/plate). Synchronized nematode larvae were also transferred to NGM plates containing *E. coli* OP50 and double distilled water (0.2 ml) as control. After incubation at 20° C for 3 days, young adult nematodes were selected for further analysis.

### Fluorescence microscopy

Experiments were performed as described before [29]. Briefly, transgenic SJL1 nematodes expressing FIB-1::GFP and treated with GL, subfractions and/or control for 3 days as described above were paralyzed by treatment with 1 mM levamisole, prior to mounting on 3% agarose gel pads. The preparations were covered with a cover slide and examined at a fixed exposure time under fluorescence microscopy (Olympus IX-70; Olympus). Whole-worm GFP levels were quantified from 30 nematodes per groups using the Image-Pro Plus software (Media Cybernetics). Worm size was evaluated by selecting a region of interest (ROI).

### Pharyngeal pumping assay

Synchronized young adult nematodes treated with water, GL (0.1–30 mg/plate), or rapamycin (400 μM) were observed under an optical DM6 B microscope equipped with a digital MC170 HD camera (Leica). Contractions in the terminal bulb of the pharynx were recorded for 1 min.

### Triglyceride quantification

Treated SJL1 *C. elegans* were collected and washed twice with PBST buffer to remove bacteria. Samples were dissolved in 0.05% Tween 20 and homogenized using TissueLyser II (Qiagen). Lysates were centrifuged and supernatants were used for triglyceride and protein quantification using commercial assay kits (Biovision; Bio-Rad). Triglyceride content was normalized against protein concentration.

### Lifespan assay

Synchronized L1 nematode larvae (n=100/group) were seeded on NGM plates (10 larvae/plate) containing *E. coli* OP50 with or without GL (2 or 20 mg/plate), sub-fractions (0.1–2 mg/plate), or rapamycin (400 μM) spread evenly on the plate, before incubation at 20° C. Live and dead nematodes were counted every other day using an optical microscope based on motility. Survival curves were determined using the Kaplan-Meier method. Worms in and outside the bacterial lawn containing GL were counted to evaluate possible attraction or repulsion by GL.

### Cell culture

Human Huh7 hepatoma cells stably-expressing RFP-LC3, GFP-LC3 and RFP-GFP-LC3 were prepared as described before [43]. Huh7 cells were maintained in Dulbecco’s modified Eagle’s medium (DMEM) containing 10% fetal bovine serum (FBS)/1% non-essential amino acids. In some experiments, cells were treated with DMEM containing FBS and 3-methyladenine (2 mM; Sigma) for 12 hrs. Human IMR-90 lung fibroblasts (American Type Culture Collection; ATCC CCL-186) were maintained in Eagle’s minimum essential medium (EMEM) containing 10% FBS. Cells were cultured in standard cell culture conditions at 37° C under 5% CO_2_.

### Cell viability

Huh7 cells or IMR-90 cells were seeded in 96-well plates at 10^4^ cells/well in 100 μl per well of culture medium and cultured for 24 hrs. Adherent cells were treated with fresh medium supplemented with either GL or the culture medium. Culture supernatants were replaced with phenol red-free medium containing 1% FBS and 10 μl of Cell Counting Kit-8 Solution (CCK-8; MedChem Express), prior to incubation for one hour. Light absorbance was measured at 450 nm using a spectrophotometer.

### Western blotting

For Western blotting in *C. elegans*, synchronized L1 larvae from the SJL1 or DA2123 genetic background were prepared and transferred to NGM plates containing *E. coli* OP50, GL, sub-fractions and/or rapamycin. After incubation at 20° C for 3 days, nematodes were collected and washed with PBS. Nematodes were pelleted by centrifugation and 40 μl of loading buffer was added. After heating at 100° C for 5 min, insoluble debris were removed by centrifugation and cell lysates were separated on a 10% sodium dodecyl sulfate-polyacrylamide gel electrophoresis (SDS-PAGE), prior to transfer onto polyvinylidene fluoride (PVDF) membranes (Millipore). After blocking with 5% (w/v) non-fat milk in PBST, blots were probed with antibodies against FIB-1 (sc-1666001, Santa Cruz Biotechnology), glyceraldehyde 3-phosphate dehydrogenase (GAPDH; 60004-1-Ig, Proteintech), GFP (GTX113617, GeneTex), or actin (sc-47778, Santa Cruz Biotechnology). Signals were detected using enhanced chemiluminescence (Millipore) according to the manufacturer’s instructions. Densitometry was done using the ImageJ software.

For human cells, Huh7 and IMR-90 cells were washed with PBS, prior to lysis in RIPA buffer (Bio Basic) with the phosphatase inhibitor PhosStop (Roche). Cell lysates were collected by centrifugation at 13,000×*g* for 10 min at 4° C. Protein concentrations were determined using the Bradford assay (Bio-Rad). Lysates were mixed with gel loading buffer (20% glycerol, 3% SDS, 3% 2-mercaptoethanol, 10 mM Tris, 0.2% bromophenol blue, pH 6.8). Lysates were boiled for 5 min, prior to separation using SDS-PAGE as above. Separated proteins were transferred to PVDF membranes. Blocking was done with 5% (w/v) bovine serum albumin (Sigma) in PBST. Membranes were incubated with rabbit antibodies raised against p-mTOR (5536), mTOR (2983), p-IRS-1 (2381), IRS-1 (2382), p-Akt (4060), Akt (4691), p-GSK-3β (5558), GSK-3β (9315), LC3B (3868), Beclin-1 (3738S), p44/42 MAPK (4695S), p-p44/42 MAPK (4370S), p38 MAPK (8690) (all from Cell Signaling Technology), p-p38 MAPK (09-272, Millipore), β-actin (66009-1-Ig, Proteintech; A1978, Sigma), or GAPDH (60004-1-Ig, Proteintech). Goat anti-rabbit IgG (AP307P, Millipore) and goat anti-mouse IgG (AP308P, Millipore) were used as secondary antibodies. Signals were detected as above.

### Transmission electron microscopy

Cells were harvested and fixed in 2.5% glutaraldehyde, 4% paraformaldehyde in 0.1 M sodium cacodylate buffer, pH 7.2, for 2 hrs at room temperature. Cells were incubated in 1% osmium tetroxide for 1 hr at room temperature, followed by dehydration in a graded series of ethanol and embedding in Epon812 resin (Electron Microscopy Sciences). Ultra-thin cell sections (70 nm) were prepared using the EM UC7 Ultramicrotome (Leica Microsystems). Ultra-thin sections were stained with uranyl acetate and Reynolds lead citrate solutions. Microscopy observations were made using a JEM-1230 transmission electron microscope (JEOL) operating at 100 kV. Autophagosomes were characterized by intracellular double-membrane vesicles containing material with homogenous electron density, whereas autolysosomes were characterized by intracellular single-membrane vesicles containing material with dense, non-homogenous electron density.

### Immunofluorescence and confocal microscopy

For immunofluorescence and confocal microscopy, cells were fixed in 4% paraformaldehyde and permeabilized using 0.1% Triton X-100 (T8787, Sigma). After extensive washing, cells were analyzed under confocal microscopy (LSM780, Zeiss). Detection of autophagosomes and autolysosomes in IMR-90 cells was done by staining with DAPRed and DALGreen (Dojindo Molecular Technologies), following the manufacturer’s guidelines. DAPRed is a hydrophobic dye that fluoresces in hydrophobic conditions found in autolysosomes and autophagosomes, while DALGreen is a hydrophobic dye that emits fluorescence in the acidic conditions of autolysosomes. Staining solution containing DAPRed and DALGreen at final concentration of 0.5 μM DAPRed and 1 μM DALGreen was added to cell monolayers, followed by incubation at 37° C for 30 min. After incubation, cells were rinsed twice with the culture medium, prior to treatment with the medium, GL or sub-fractions. Stained cells were further incubated at 37° C for 16 hrs, prior to confocal microscopy observation.

### Statistical analysis

Data from two to four replicate experiments are presented as means ± standard errors of the mean (SEM). Mean differences were assessed using unpaired Student’s *t*-test. Data sets that involved more than two groups were assessed by one-way analysis of variance (ANOVA) followed by Dunnett’s least significant difference test. *P* values smaller than 5% were considered statistically significant. Survival differences were tested for significance using the log-rank test.

## Supplementary Material

Supplementary Figures

Supplementary Tables
