# Supplementary material for: Ganoderma lucidum stimulates autophagy-dependent longevity pathways in Caenorhabditis elegans and human cells
Source: Aging (Albany NY). 2021 May 20;13(10):13474–95. doi: 10.18632/aging.203068 (PMC8202889; doi:10.18632/aging.203068)
Supplement: Supplementary Figures [file aging-13-203068-s001.pdf]

SUPPLEMENTARY FIGURES

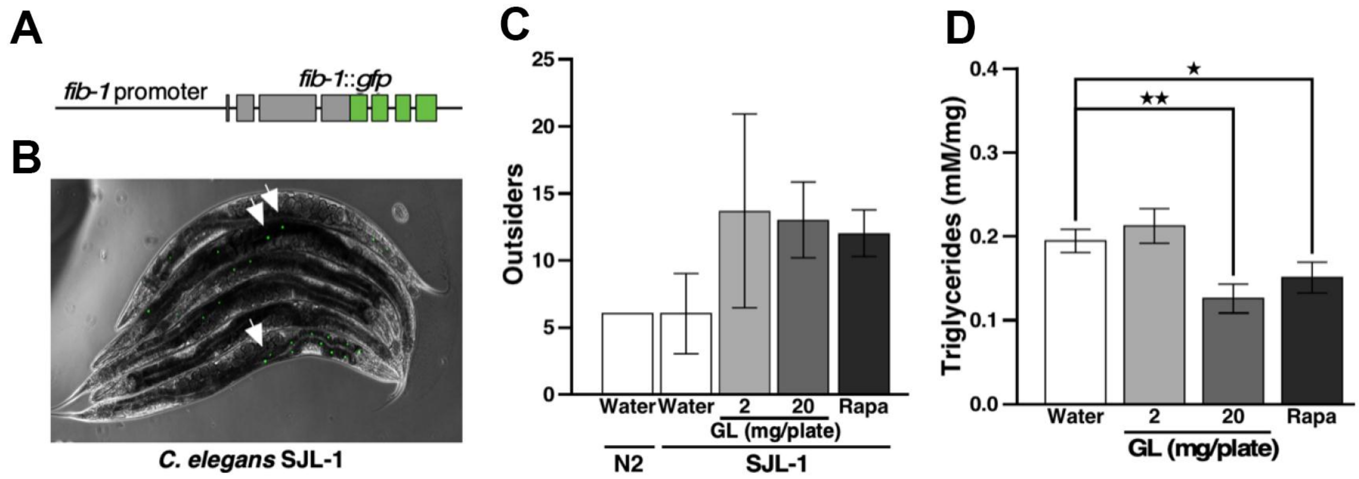

Supplementary Figure 1. FIB-1-GFP transgene construct and effects of *G. lucidum* on *C. elegans* repulsion and triglyceride content. (**A**) Transgene construct of fused FIB-1::GFP expressed in SJL1 nematodes. *fib-1* expression is driven by its native promoter. (**B**) Merged bright-field and fluorescence microscopy images of SJL1 nematodes showing the expression of FIB-1::GFP as green dots (denoted by arrows). (**C**) Effects of the water extract of *G. lucidum* (GL) on nematodes found in the bacterial lawn. The number of outsiders that escaped from bacterial lawn containing water, GL or rapamycin (Rapa) on NGM agar plates was determined after 3 days. (**D**) Triglyceride content of worms after treatment with water, GL or Rapa for 3 days.*p<0.05; **p<0.01.

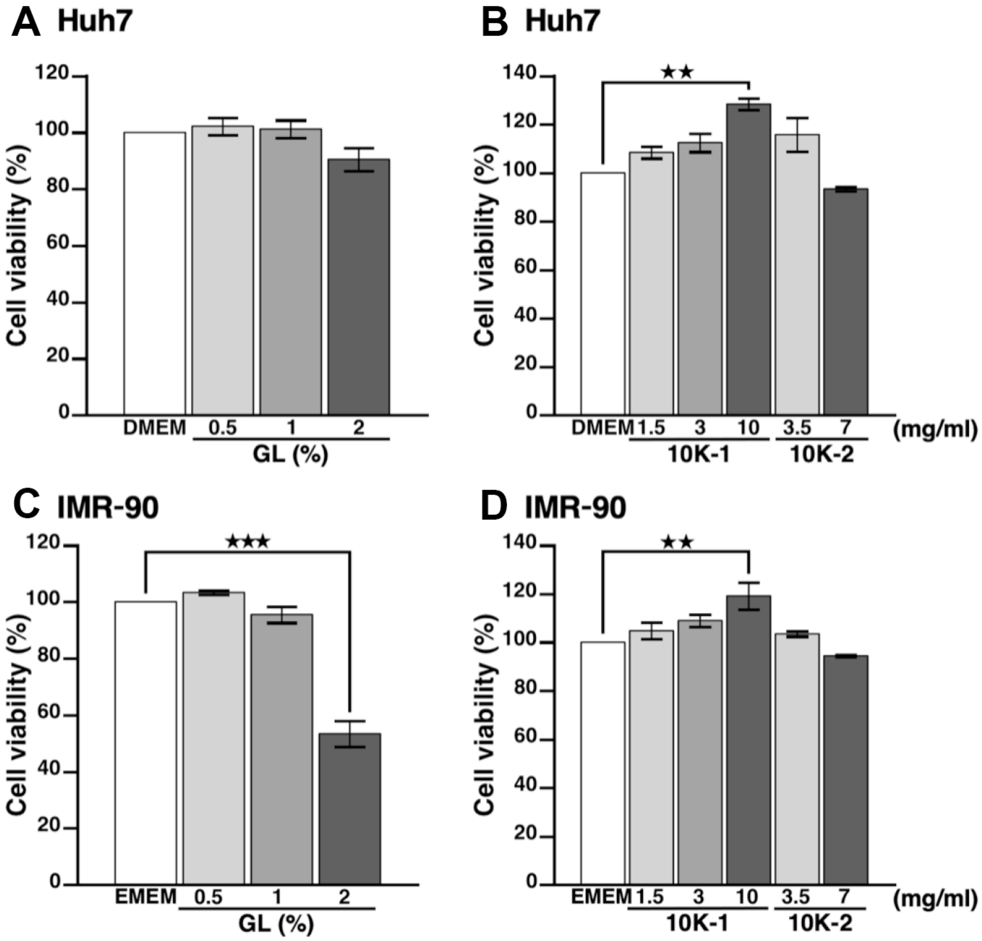

Supplementary Figure 2. Effects of *G. lucidum* and sub-fractions on cell viability. (**A**, **B**) Effects of the water extract of *G. lucidum* (GL) and sub-fractions 10K-1 and 10K-2 on Huh7 cell viability. Human Huh7 hepatocytes were treated with Dulbecco's modified Eagle's medium (DMEM), GL or sub-fractions for 24 hrs. Cell viability was monitored using the CCK-8 assay. (**C**, **D**) Effects of GL and sub-fractions on IMR-90 cell viability. Human IMR-90 lung fibroblasts cultured in Eagle's minimum essential medium (EMEM) were treated as above. Data are shown as means ± standard error of the mean (SEM) of four independent experiments. $^{**}p < 0.01$, $^{***}p < 0.001$.

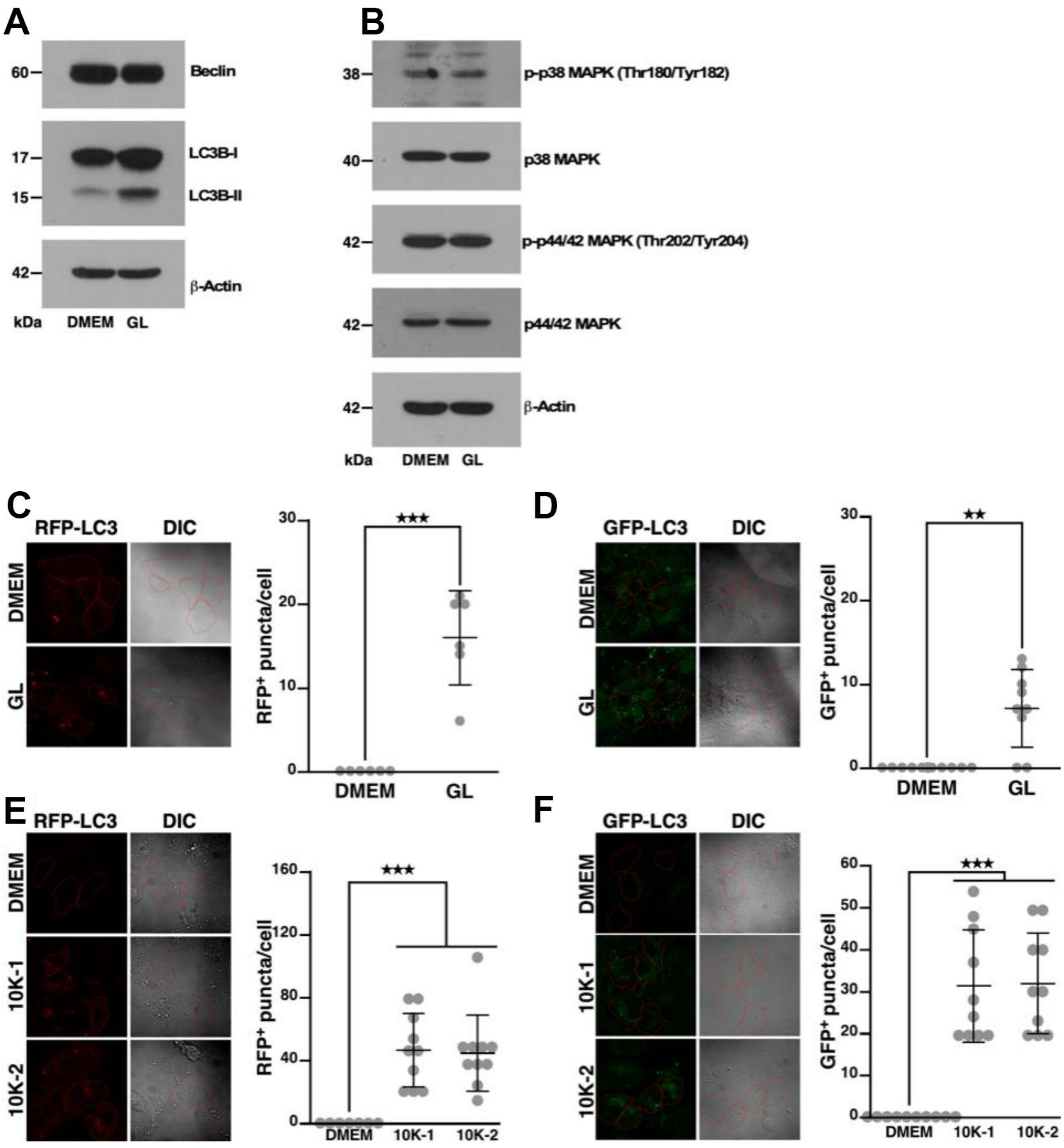

Supplementary Figure 3. Effects of *G. lucidum* on Beclin-1 and MAPKs in human cells. (**A**, **B**) Huh7 cells were treated with *G. lucidum* (GL; 1 mg/ml) or DMEM for 12 hrs, prior to Western blot analysis of Bectin-1, LC3B-I, LC3B-II and MAPKs. Protein expression was normalized against actin. (**C**–**F**) Effects of GL and sub-fractions 10K-1 and 10K-2 on RFP-LC3 and GFP-LC3 fluorescent puncta in Huh7 cells. Cells expressing RFP-LC3 and GFP-LC3 were cultured in Dulbecco's modified Eagle's medium (DMEM). The cells were treated with GL (1%), 10K-1 (1 mg/ml), or 10K-2 (1 mg/ml) for 24 hrs, prior to fluorescence microscopy analysis. In differential interference contrast (DIC) images, cells were delineated in red for clarity. Data are shown as means ± standard error of the mean (SEM) of four independent experiments. $^{**}p < 0.01$, $^{***}p < 0.001$.

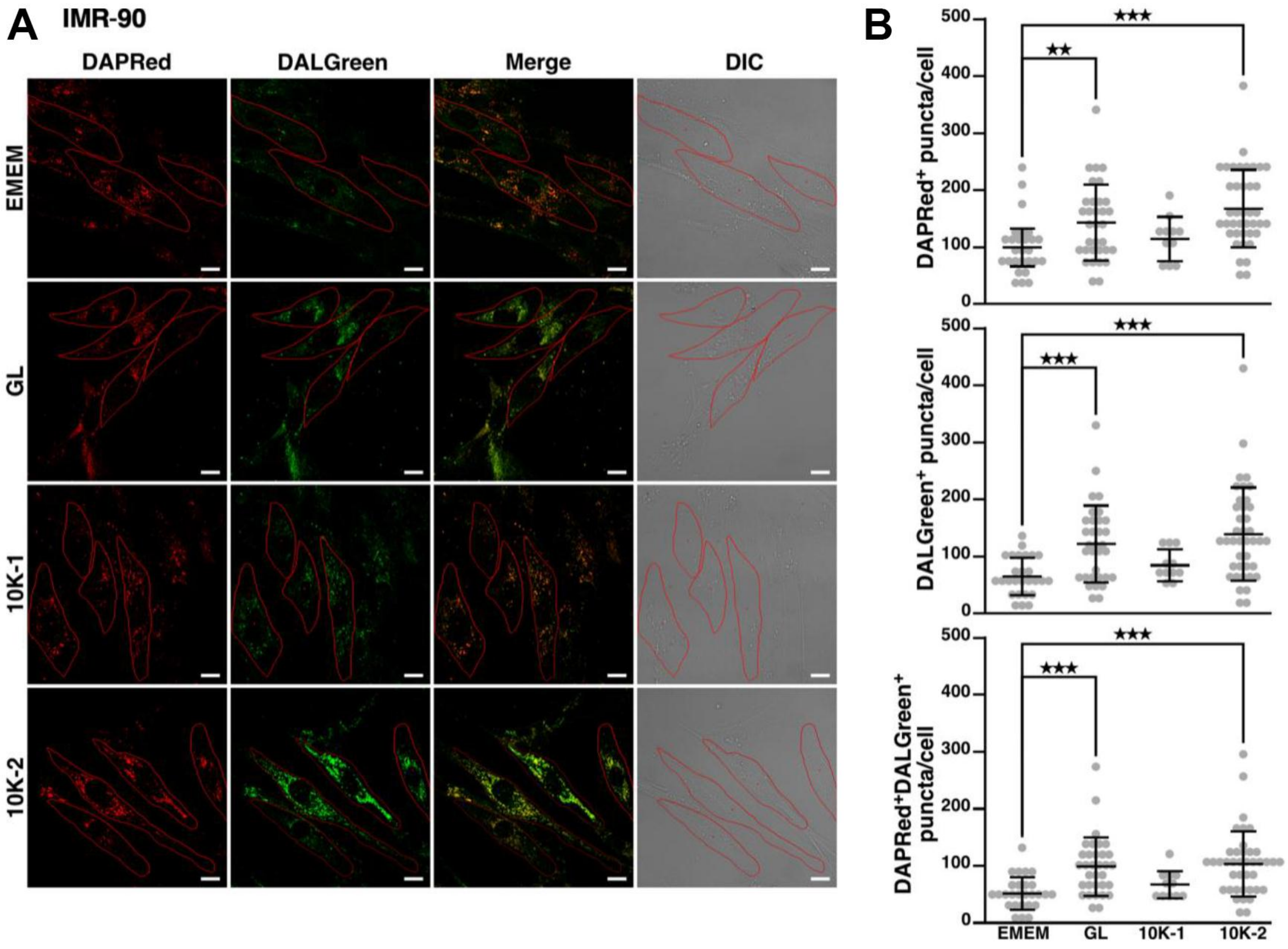

Supplementary Figure 4. *G. lucidum* and sub-fractions induce autophagy in IMR-90 cells. (**A**) Confocal microscopy observations of IMR-90 human lung fibroblasts treated with the water extract of *G. lucidum* (GL) or sub-fractions (10K-1 or 10K-2). Cells cultured in Eagle's miminum essential medium (EMEM) were stained with DAPRed and DALGreen. Autophagosomes and autolysosomes are stained by DAPRed, while autolysosomes are stained by DALGreen. Cells were cultured with GL (1%), 10K-1 (1.5 mg/ml), or 10K-2 (3.5 mg/ml) for 16 hrs, prior to observation under confocal microscopy. In differential interference contrast (DIC) images, cells were delineated in red. Scale bars: 20 µm. (**B**) Quantification of fluorescent puncta. **p<0.01; ***p <0.001.
