# Supplementary material for: Ganoderma lucidum stimulates autophagy-dependent longevity pathways in Caenorhabditis elegans and human cells
Source: Aging (Albany NY). 2021 May 20;13(10):13474–95. doi: 10.18632/aging.203068 (PMC8202889; doi:10.18632/aging.203068)
Supplement: Supplementary Tables [file aging-13-203068-s002.pdf]

SUPPLEMENTARY TABLES

Supplementary Table 1. Summary of lifespan assays performed in this study.

Nematode strain	Treatment	Median lifespan ± SEM	p value	Maximum lifespan ± SEM	p value	Trials
SJL1 (FIB-1::GFP)	Control water	13.0 ± 2.8		18.8 ± 1.6		2
SJL1 (FIB-1::GFP)	Rapamycin 400 µM	16.0 ± 2.8	<0.0001	24.9 ± 2.6	0.07	2
SJL1 (FIB-1::GFP)	GL 2 mg/plate	18.5 ± 6.3	<0.0001	25.7 ± 0.8	0.02	2
SJL1 (FIB-1::GFP)	GL 20 mg/plate	15.5 ± 2.1	<0.0001	28.9 ± 2.3	0.02	2
N2 (wild-type)	Control water	13.5 ± 0.7		21.5 ± 1.5		2
N2 (wild-type)	Rapamycin 400 µM	15	<0.0001	26		1
N2 (wild-type)	GL 2 mg/plate	14 ± 1.4	<0.0001	24 ± 3	0.26	2
VC893 (*atg18*)	Control water	10 ± 0		15 ± 1		2
VC893 (*atg18*)	Rapamycin 400 µM	10	0.7	16		1
VC893 (*atg18*)	GL 2 mg/plate	10 ± 0	0.57	14.25 ± 0.25	0.27	2
CF1037 (*daf-16*)	Control water	15 ± 1		18.75 ± 1.25		2
CF1037 (*daf-16*)	Rapamycin 400 µM	16	<0.0001	21		1
CF1037 (*daf-16*)	GL 2 mg/plate	16 ± 0	0.2	19.25 ± 1.75	0.41	2
VC199 (*sir-2.1*)	Control water	13.5 ±0.5		21.25 ± 2.25		2
VC199 (*sir-2.1*)	Rapamycin 400 µM	15	<0.0005	22		1
VC199 (*sir-2.1*)	GL2 mg/plate	12.5 ± 0.5	0.42	22.5 ± 3.5	0.39	2
SJL1 (FIB-1::GFP)	Control water	15		23		1
SJL1 (FIB-1::GFP)	Rapamycin 400 µM	17	0.033	24.5		1
SJL1 (FIB-1::GFP)	10K-1, 0.1 mg/plate	15	0.12	21		1
SJL1 (FIB-1::GFP)	10K-1, 1 mg/plate	15	0.99	24		1
SJL1 (FIB-1::GFP)	Control water	15		24.5		1
SJL1 (FIB-1::GFP)	Rapamycin 400 µM	17	0.081	26		1
SJL1 (FIB-1::GFP)	10K-2, 0.2 mg/plate	15	0.53	25.5		1
SJL1 (FIB-1::GFP)	10K-2, 2 mg/plate	17	0.046	25		1

Maximum lifespan was calculated from the 10% of worms with the longest lifespan. Abbreviations: FIB-1, fibrillarin; GFP, green fluorescent protein; SEM, standard error of the mean; GL, *Ganoderma lucidum* (water extract).

Supplementary Table 2. Monosaccharide composition of the water extract of *G. lucidum* (GL) and sub-fractions 10K-1 and 10K-2.

Monosaccharide	GL (%)	10K-1 (%)	10K-2 (%)
Arabinose	4.2	2.3	7.5
Fucose	2.4	4.3	0.8
Fructose	16.6	6.8	30.4
Galactose	9.0	9.3	9.1
Glucose	33.2	33.2	31.4
Mannose	31.5	43.7	13.2
Ribose	3.1	0.6	7.5

Monosaccharide content was determined using high-performance ion chromatography with pulsed amperometric detection.

Supplementary Table 3. Monosaccharide molar ratio of the water extract of *G. lucidum* (GL) and sub-fractions 10K-1 and 10K-2.

Monosaccharide	GL	10K-1	10K-2
Arabinose	0.15	0.06	0.29
Fucose	0.08	0.11	0.03
Fructose	0.50	0.16	0.97
Galactose	0.27	0.21	0.29
Glucose	1.00	0.76	1.00
Mannose	0.95	1.00	0.42
Ribose	0.11	0.02	0.28

Analysis was performed using high-performance ion chromatography with pulsed amperometric detection. Molar ratio was calculated by dividing the molarity of each monosaccharide by the highest molarity for the given specimen.

Supplementary Table 4. Protein content of the water extract of *G. lucidum* (GL) and sub-fractions 10K-1 and 10K-2.

	GL(%)	10K-1(%)	10K-2(%)
Protein	1.73 ± 0.03	3.85 ± 0.04	0.41 ± 0.04

Analysis was done using the Bradford colorimetric assay. Protein content was calculated by dividing the protein concentration measured by the concentration of dry weight matter (w/v) for each sample, followed by multiplication by 100.

Supplementary Table 5. Molecular weight analysis of 10K-1 and 10K-2 sub-fractions.

	10K-1	10K-2
Sample concentration (mg/ml)	3.14	4.08
Peak retention volume (ml)	19.54	22.78
Mn: number average molecular weight (Da)	314364	9089
Mw: weight average molecular weight (Da)	758028	83230
Mp: molecular weight of the highest peak (Da)	150993	45114
Mw/Mn ratio (polydispersity)	2.41	9.16

Analysis was done using gel permeation chromatography.
